# Mechanisms underlying the therapeutic effects of *Semen cuscutae* in treating recurrent spontaneous abortion based on network pharmacology and molecular docking

**DOI:** 10.3389/fmolb.2024.1282100

**Published:** 2024-05-30

**Authors:** Wenfei Zheng, Manshu Lei, Yao Yao, Jingqiong Zhan, Yiming Zhang, Quan Zhou

**Affiliations:** Department of Gynecology and Obstetrics, The First College of Clinical Medical Science, China Three Gorges University/Yichang Central People’s Hospital, Yichan, China

**Keywords:** immune-inflammatory, *Semen cuscutae*, recurrent spontaneous abortion, network pharmacology, molecular docking, tumor necrosis factor-alpha inhibitor

## Abstract

**Background:** This paper aims to analyse the active components of *Semen cuscutae* (SC) by network pharmacology and screen the most stable compounds with tumour necrosis factor-alpha (TNF-α) by molecular docking to explore the mechanisms of SC treatment of recurrent spontaneous abortion (RSA) and provide a theoretical basis for drug development.

**Methods:** The active compounds of SC and the potential inflammatory targets of RSA were obtained from the Traditional Chinese Medicine Systems Pharmacology database and GeneCards, respectively. The RSA-SC target gene interaction network was obtained and visualized using the STRING database and Cytoscape software. GO and KEGG pathway enrichment analyses were obtained from DAVID to further explore the RSA mechanism and therapeutic effects of SC. Interactions between TNF-α and drugs were analysed by molecular docking. Treatment of human trophoblast cells with sesamin and TNF-α was carried out to detect their proliferative and apoptotic abilities, and WB assay was carried out to detect EGFR, PTGS2, and CASP3 protein expression.

**Results:** Ten compounds and 128 target genes were screened from *SC*, of which 79 overlapped with RSA target inflammatory genes, which were considered potential therapeutic targets. Network pharmacological analysis showed that sesamin, matrine, matrol, and other SC compounds had a good correlation with the inflammatory target genes of RSA. Related genes included PGR, PTGS1, PTGS2, TGFB1, and CHRNA7. Several signalling pathways are involved in the pathogenesis of RSA, such as the TNF-α signalling pathway, HIF-1 signalling pathway, oestrogen signalling pathway, proteoglycans in cancer cells, and FoxO signalling pathway. Molecular docking results suggested that sesamin was the most suitable natural tumour necrosis factor inhibitor (TNFi). Sesamin can promote proliferation and inhibit apoptosis in human trophoblasts by downregulating EGFR and CASP3 expression and upregulating PTGS2 expression.

**Conclusion:** Our findings play an important role and basis for further research into the molecular mechanism of SC treatment of RSA and drug development of TNFi.

## 1 Introduction

Recurrent spontaneous abortion (RSA), a common pregnancy complication, is defined as three or more clinically confirmed pregnancy failures, which occurs in 12% of pregnant women (Wang et al., 2021a) and places a heavy burden on families and society. A large number of studies confirm that RSA significantly affects the quality of life of women and their partners, increasing anxiety and depression in women with recurrent abortion (Côté-Arsenault et al., 2020; Gao et al., 2020). In addition, RSA is a risk factor for cardiovascular disease later in life (Wu et al., 2019; McNestry et al., 2023).

The causes of recurrent abortion are complex, and the treatment options are mainly clinical psychological support and empiric therapy, including antithrombotic therapy and anti-immunotherapy, using aspirin, heparin, intravenous immunoglobulin (IVIG), and other therapies, such as glucocorticoids, intra-lipid infusion, hormone supplementation with progesterone or dydrogesterone, granulocyte colony-stimulating factor, and lymphocyte immunotherapy. However, the efficacy of these drugs is controversial, and they also have many side effects, including local allergy, liver damage, renal insufficiency, thrombocytopenia, osteoporosis, fatigue, and lethargy, which confuse RSA patients (Guo et al., 2018; Bai et al., 2021). Therefore, it is essential to investigate effective and natural products to reduce pregnancy loss in patients with RSA.

In recent years, traditional Chinese medicine has become a research hotspot as a popular complementary treatment of Western medicine for recurrent spontaneous abortion patients, with satisfactory curative effects, high safety, and reliability. *Cuscuta* (Chinese name: Tusizi), the dried seeds of southern and Chinese dodder, has been used for many years to cure abortion (Maimaiti et al., 2021). *Semen cuscutae* (SC), which is used as the core component of many classical herbal formulae, has functions of nourishing the kidney and liver and anti-inflammatory, antibacterial, antioxidant, and antiulcer properties (Liao et al., 2014; Ding et al., 2018).

It has been shown that lignans and flavonoids from *Cuscuta sinensis* extract could inhibit the release of NO and the inflammatory factors PGE2, tumour necrosis factor-α (TNF-α), and IL-6, thus suppressing the inflammatory response, and lignans could inhibit the expression level of cellular iNOS genes (Mao, 2019; Suyi, 2021). Although SC is widely used for the treatment of abortion and RSA, the exact mechanisms still need to be further elucidated.

TNF-α plays an important role in cell proliferation, differentiation, apoptosis, immune regulation, and inflammation induction and has a certain lethality to tumour cells, trophoblasts, and embryonic tissues (Yang et al., 2020; Abdellatif et al., 2022). Some research studies have confirmed that the serum level of TNF-α is higher in patients with recurrent abortion, suggesting that increased TNF-α levels may lead to abortion (Arslan et al., 2004). By downregulating TNF-α to promote IL-6 expression, the SC extract can reduce maternal and foetal immune tolerance and maintain pregnancy continuation (Yang et al., 2020). Recently, TNF-α has attracted more attention because of its important role in the inflammatory immune process, and inhibiting its biological activity may be used as a new direction for drug development.

Network pharmacology is an effective method for drug discovery. This paper aims to analyse the active components of SC by network pharmacology and screen the most stable compounds with TNF-α by molecular docking to explore the mechanism of SC treatment of RSA and provide a theoretical basis for drug development.

## 2 Materials and methods

### 2.1 Database website

The databases used in this paper are shown in Table 1.

**TABLE 1:** Database website used in this study.

Database	Website
TCMSP	http://lsp.nwu.edu.cn/tcmsp.php
GeneCards	https://www.genecards.org/
Venny	http://bioinformatics.psb.ugent.be/webtools/Venn/
STRING	https://string-db.org/
UniProt	https://www.uniprot.org/
CB-Dock	http://clab.labshare.cn/cb-dock/php/

### 2.2 Prediction of SC-associated target genes and their intersection on RSA

The components of SC were obtained from the Traditional Chinese Medicine Systems Pharmacology and Analysis Platform (TCMSP; https://tcmspw.com/index.php) The TCMSP is a Chinese herbal medicine system data platform on the drug–disease–target relationship, involving the pharmacokinetic properties of natural compounds, which includes oral bioavailability, intestinal epithelial permeability, drug similarity, blood–brain barrier, and water solubility (Ru et al., 2014).

According to literature reports and pharmacokinetic parameters, drug absorption, distribution, metabolism, and excretion (ADME) are important factors influencing drug bioactivity. According to the TCMSP recommendations, compounds with an oral bioavailability (OB) of 30% are well-absorbed and metabolised slowly after oral administration. A drug similarity (DL) of 0.18 is chemically suitable for drug development (Xie et al., 2018). Therefore, according to the relevant screening criteria (OB ≥ 30% and DL ≥ 0.18), a total of 11 compounds and their target proteins were obtained from the TCMSP database. RSA-related target genes were obtained from GeneCards. The combination target genes of SC treatment for RSA were obtained from Venny.

### 2.3 Construction of the protein–protein interaction (PPI) network and functional enrichment analysis of related genes

STRING is a database for the construction of protein–protein interactions (PPIs) (Szklarczyk et al., 2021). The combination target genes were entered into the STRING database for further analysis. The screening criteria were as follows: *Homo sapiens* and a confidence score greater than 0.4. The *SC*–compound–target RSA network was visualised through Cytoscape software (version 3.6.1) (Zuo et al., 2018). The R software “clusterProfiler” and “Pathview” packages were used to perform GO and KEGG pathway analysis and visualization.

### 2.4 Molecular docking technology

The active components selected from SC were ligated to TNF-α receptors using CB-Dock. CB-Dock is an online server that can automatically identify binding sites without predictive information and then dock using AutoDock Vina according to the query results, with a success rate of about 70%, which is better than that of other commonly used blind docking tools (Trott and Olson, 2010). The PDB file for the TNF-α (protein ID: 2az5) was searched in the UniProt database, while the active components were downloaded from the TCMSP. They were deposited on CB-Dock. The Vina scores were used to evaluate the binding activity. The lower the Vina score, the more stable the binding activity.

### 2.5 Experimental verification

#### 2.5.1 HTR-8 culture

Human trophoblast cells (HTR-8; Saierbio, Tianjin, China) were grown in Dulbecco’s modified Eagle’s medium (Gibco, United States) plus 10% foetal bovine serum (FBS), 100 U/mL penicillin, and 100 U/mL streptomycin. HTR-8 cells were grown in an environment of 5% CO_2_ at 37°C.

#### 2.5.2 Cell counting kit-8 assay

Cell counting kit-8 (CCK-8) assay was performed according to the manufacturer’s protocol. HTR-8/SVneo cells were seeded into 96-well plates at a density of 3 × 103 cells/well, and the complete culture solution containing different concentrations of sesamin (:0, 20, 40, 60, 80, 100, 120, 140, 160, 180, and 200 μmol) and TNF-α (0, 10, 20, 30, and 40 pg/mL) was replenished to 100 μL in each well. After 24 h, 100 µL of RPMI 1640 Medium and 10 µL of CCK-8 reagent (Blue Sky Biology, China) were added to each well and incubated at 37°C for 2 h. The relative proliferation ability was measured at a wavelength of 450 nm.

#### 2.5.3 Flow cytometry

Apoptotic levels were examined using Annexin V-FITC/PI double-staining apoptosis detection kits (Yeasen Biotechnology, China). HTR-8 cells were digested by trypsin and centrifuged for 5 min at 4°C. In dark condition at 4°C, the cell suspension was stained by 5 µL Annexin V-FITC for 15 min and 10 µL PI for 5 min. Afterward, the HTR-8 cells were detected through flow cytometry (FCM) (BD, United States) within 1 h.

#### 2.5.4 Western blotting

HTR-8 cells were washed twice with ice-cold PBS. Total protein was extracted from cells using RIPA lysis buffer (Saierbio, Tianjin, China). Cells were incubated with RIPA lysis buffer under shaking and cracking at 4°C for 30 min. The supernatants were removed, and the protein concentrations were determined using a BCA kit (Saierbio, Tianjin, China). Then, 6 μL of denatured protein was separated on 10% SDS-PAGE gels and transferred onto PVDF membranes. After blocking in 5% non-fat milk at room temperature for 2 h, the membranes were incubated with primary antibodies at 4°C overnight. The primary antibodies used were monoclonal rabbit anti-EGFR (Saierbio, Tianjin, China), anti-CASP3 antibody (Saierbio, Tianjin, China), and anti-PTGS2 antibody (Saierbio, Tianjin, China). A GAPDH antibody (Saierbio, Tianjin, China) was used as a control for normalizing protein expression. On the second day, the membranes were incubated with a secondary antibody (goat anti-rabbit immunoglobulin G H&L) (HRP) (Blue Sky Biology, China) at room temperature for 1.5 h and washed four times with 1 × PBS for 5 min. The band intensities were analysed using Champ Gel 5000 Gel Imaging Analyser (e145116) (SageCreation, China).

### 2.6 Statistical analysis

Data analyses were conducted using statistical software SPSS 26.0 (IBM Corp., Armonk, NY, United States) and GraphPad Prism 6.0 (GraphPad Software Inc.) and presented as mean ± standard deviations (SDs). A *t*-test was used for comparisons between two groups. The results were considered significant when *p* < 0.05. Each experiment was repeated three times.

## 3 Results

### 3.1 Analysis of *Semen cuscutae* compounds

According to the screening criteria, a total of 11 compounds were obtained from dodder. The specific information is given in Table 2.

**TABLE 2:** Bioactive compounds of *Semen cuscutae*.

No.	Molecule ID	Molecule name	Molecular weight	OB (%)	DL
1	MOL001558	Sesamin	354.38	56.55	0.83
2	MOL000184	NSC63551	412.77	39.25	0.76
3	MOL000354	Isorhamnetin	316.28	49.60	0.31
4	MOL000358	Beta-sitosterol	414.79	36.91	0.75
5	MOL000422	Kaempferol	286.25	41.88	0.24
6	MOL005043	Campest-5-en-3beta-ol	400.76	37.58	0.71
7	MOL005440	Isofucosterol	412.77	43.78	0.76
8	MOL005944	Matrine	248.41	63.77	0.25
9	MOL006649	Sophranol	264.41	55.42	0.28
10	MOL000953	CLR	386.73	37.87	0.68
11	MOL000098	Quercetin	302.25	46.43	0.28

### 3.2 Drug–disease–compound–target gene network

**FIGURE 1:**
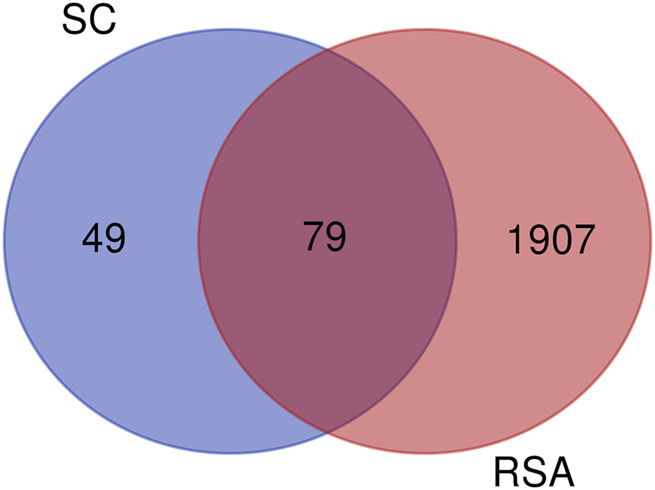
Venn diagram of 79 target genes: potential targets for *Semen cuscutae* (SC) treatment of recurrent spontaneous abortion (RSA).

**FIGURE 2:**
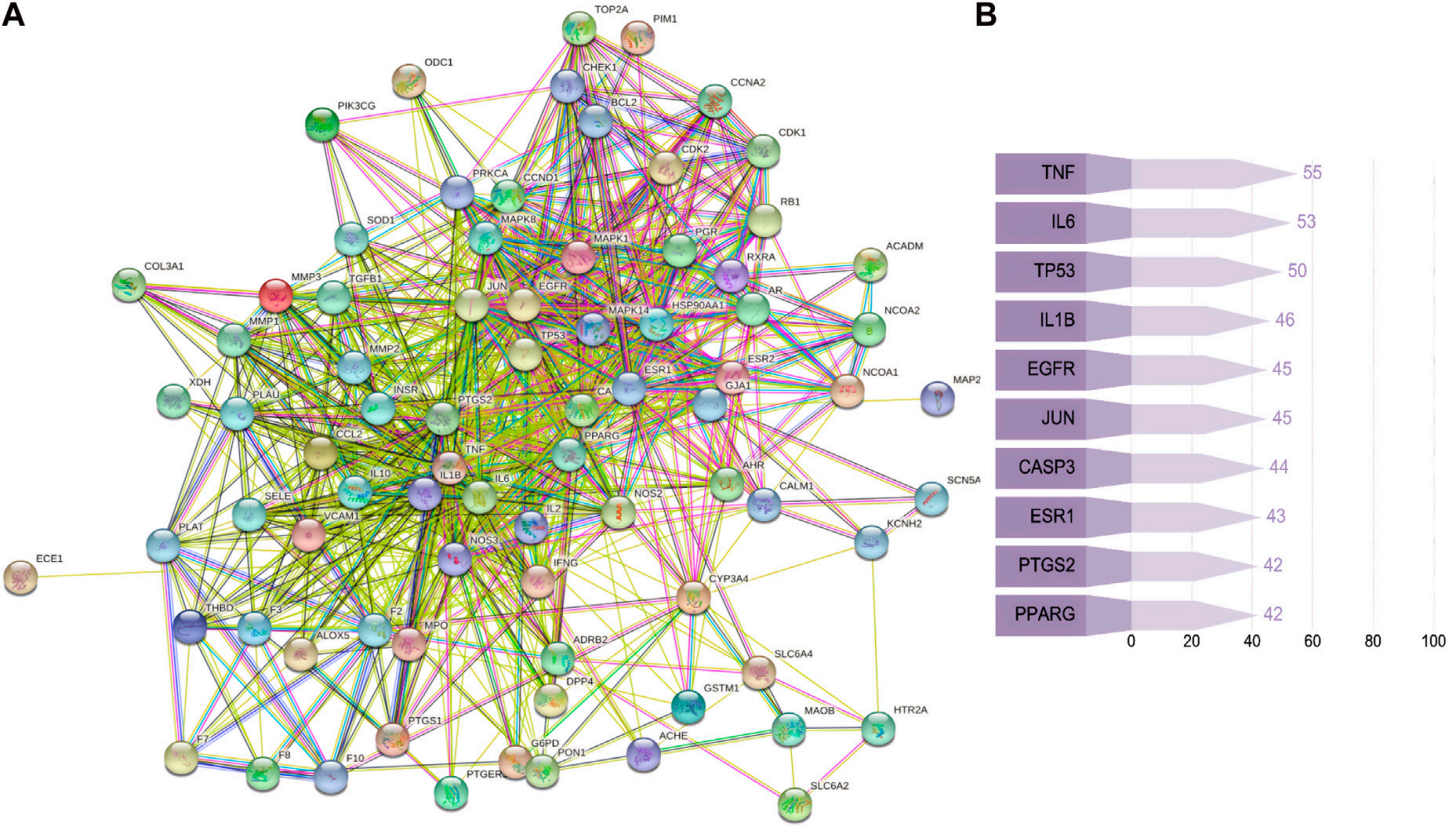
Potential target genes and protein–protein interaction (PPI) network map of SC therapy for RSA. **(A)** PPI network map of 79 target genes, and **(B)** list of the top 10 genes of the PPI network map.

**FIGURE 3:**
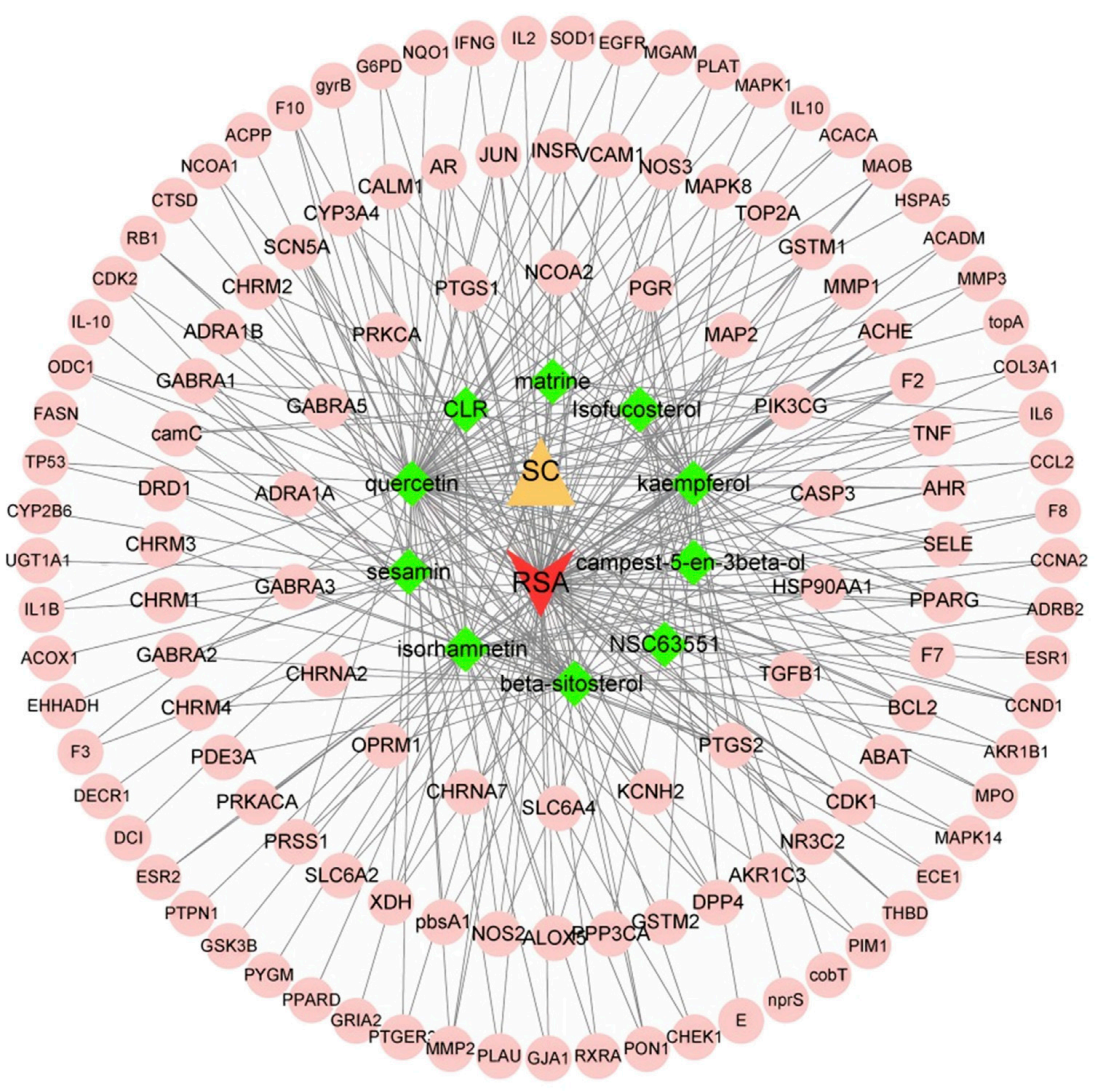
S*C*-RSA–compound–potential target gene network map. The yellow triangle represents SC, red v represents RSA, green diamonds denote the active ingredients of SC, and pink circles indicate 79 overlapping target genes.

A total of 128 target genes corresponding to 10 active components of SC were obtained from the TCMSP database (sophranol had no direct targets). Similarly, 1,986 target genes of RSA were obtained by searching the GeneCards database excluding duplicate genes. A total of 79 potential target genes were obtained by crossing RSA and SC target genes using Venny software (Figure 1). The PPI network map was obtained by importing 79 target genes into the STRING database, which was visualised by Cytoscape software (Figure 2A). The top 10 targets were found by further analysis, namely, TNF, IL-6, TP53, IL1B, EGFR, JUN, CASP3, ESR1, PPARG, and PTGS2 (Figure 2B). The above results were entered into Cytoscape to obtain an *SC*-RSA compound gene network (Figure 3).

### 3.3 Validation through the GEO database

**FIGURE 4:**
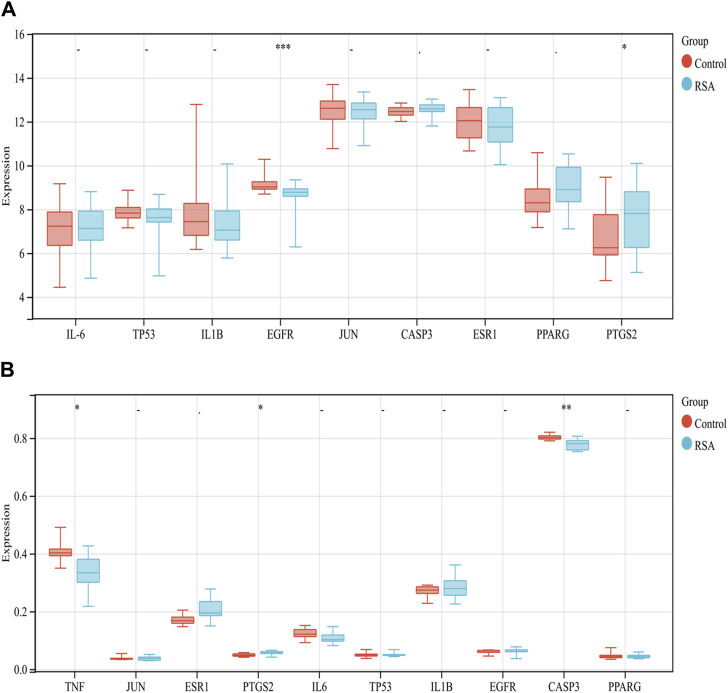
Validation of gene expression in GEO data (∗*p* < 0.05, ∗∗*p* < 0.01, and ∗∗∗*p* < 0.001). **(A)**: GSE165004 was used to verify the critical gene levels **(B)**: GSE43256 was used to verify the critical gene levels.

Databases GSE165004 and GSE43256 were selected from the GEO database to validate the above hub genes. GSE165004 includes endometrial samples from 24 RPA patients and 24 control patients on days 19–21 of menstruation. TNF is not addressed in this dataset. The expression of other genes is shown in Figure 4A. Only the expression of EGFR (*p* < 0.001) and PTGS2 (*p* < 0.05) was statistically differentiated. GSE43256 includes first-trimester placental villus samples from karyotypically normal miscarriages from recurrent miscarriage patients (N = 10) and chromosomally normal elective terminations (N = 10). As shown in Figure 4B, there is a statistically significant difference in the expression of TNF (*p* < 0.05), PTGS2 (*p* < 0.05), and CASP3 (*p* < 0.01).

### 3.4 Enrichment analysis of GO and KEGG pathways

**FIGURE 5:**
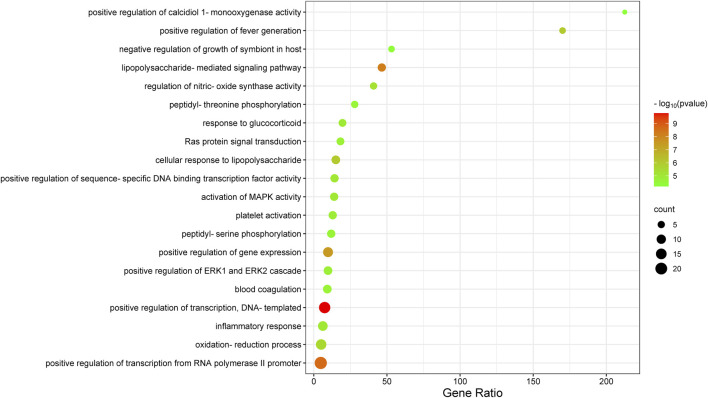
Top 20 biological functions.

A total of 57 biological function terms were obtained by GO enrichment analysis, with a threshold value of *p* < 0.05. The top 20 catalogues were selected for scatter plots (Figure 5). The biological functions of these genes are mainly positive regulation of RNA polymerase II promoter transcription, positive regulation in transcription, DNA templating, oxidation–reduction process, positive regulation in gene expression, and inflammatory response.

**FIGURE 6:**
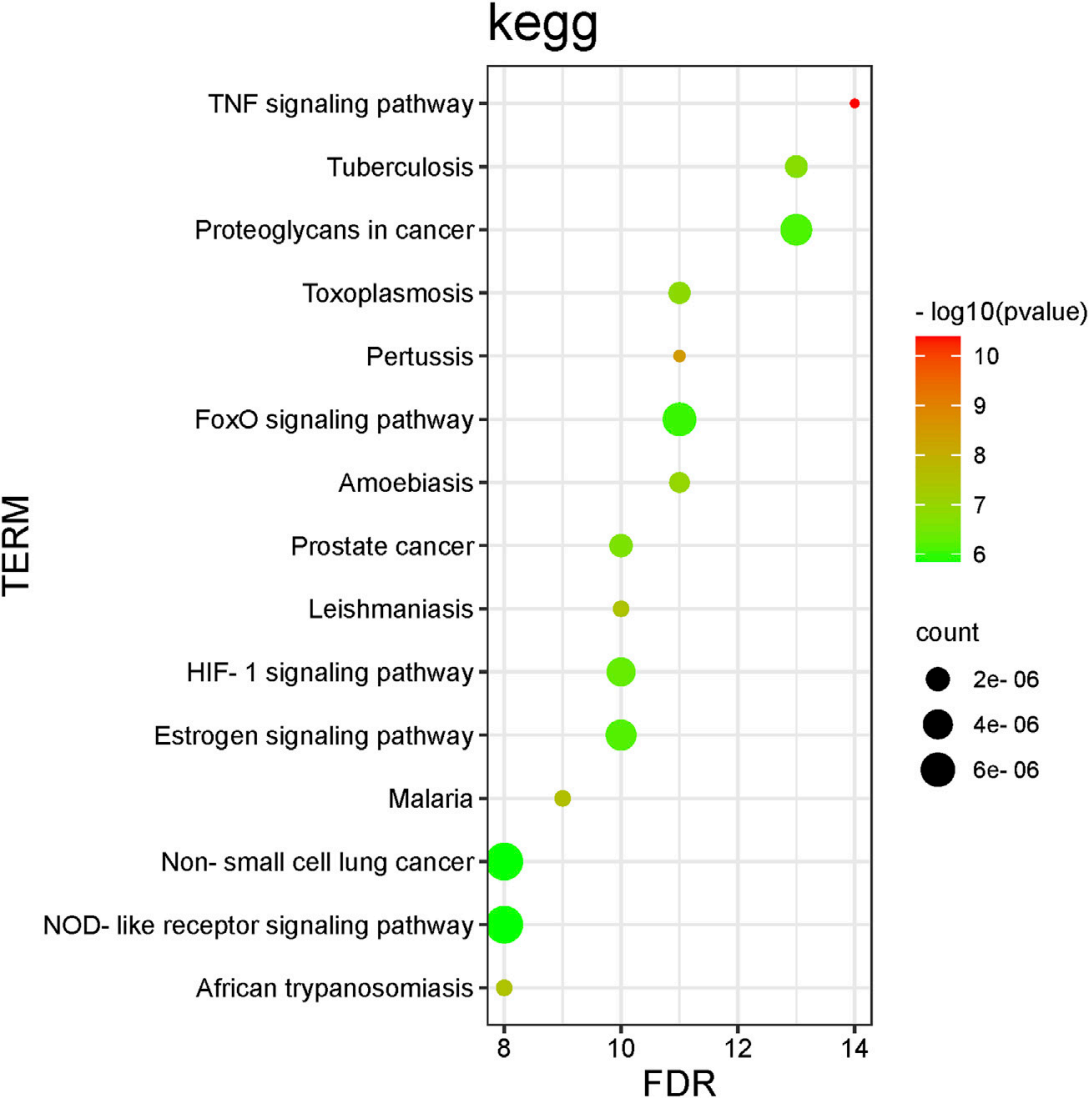
Top 15 signalling pathways for KEGG enrichment of core targets.

**FIGURE 7:**
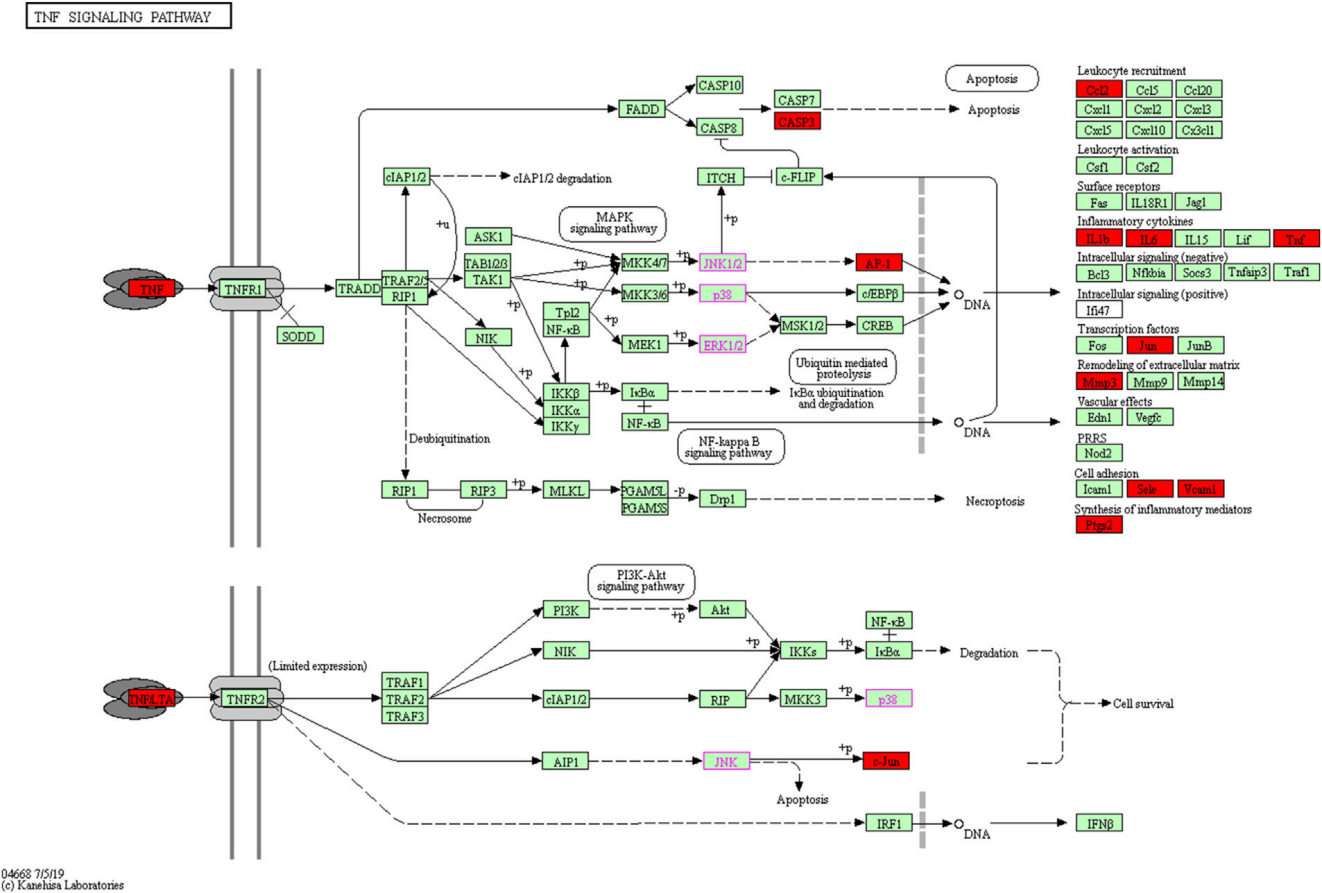
Core target of the tumour necrosis factor (TNF) signalling pathway. Red represents SC action targets in the network, and arrows indicate upstream and downstream relationships between genes.

To further understand the mechanism of SC in RSA treatment, 82 pathways were obtained by KEGG pathway enrichment analysis (*p* < 0.05). The scatter plot shows the first 15 essential signalling pathways (Figure 6), some of which are strongly related to RSA, for example, TNF signalling pathway, HIF-1 signalling pathway, estrogen signalling pathway, FoxO signalling pathway, and NOD-like receptor signalling pathway. In addition, the signalling pathway of vital TNF-α is shown in Figure 7.

### 3.5 Binding between TNF-α and active ingredients

**FIGURE 8:**
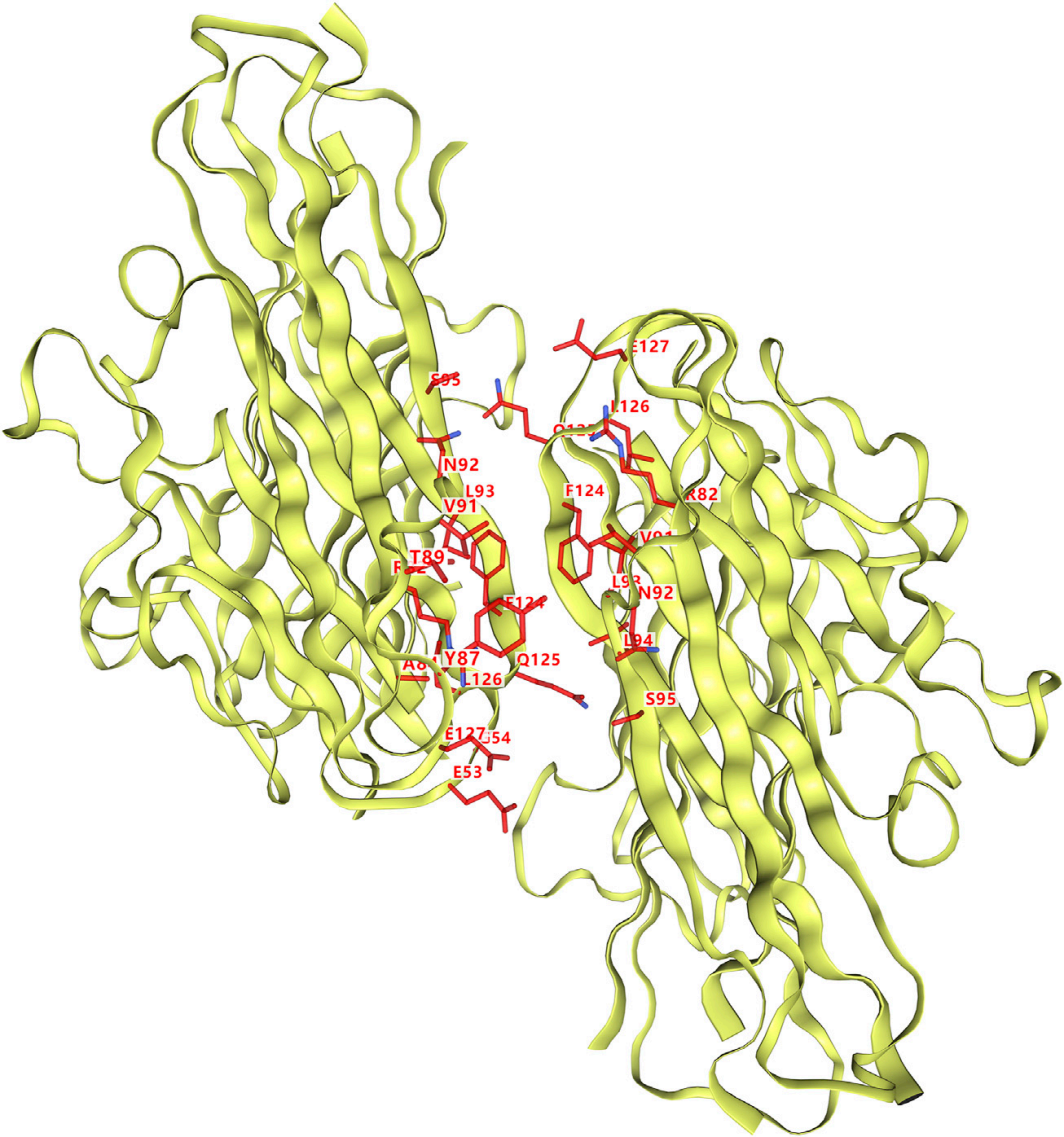
Three-dimensional structure of TNF-α and the site of binding.

**FIGURE 9:**
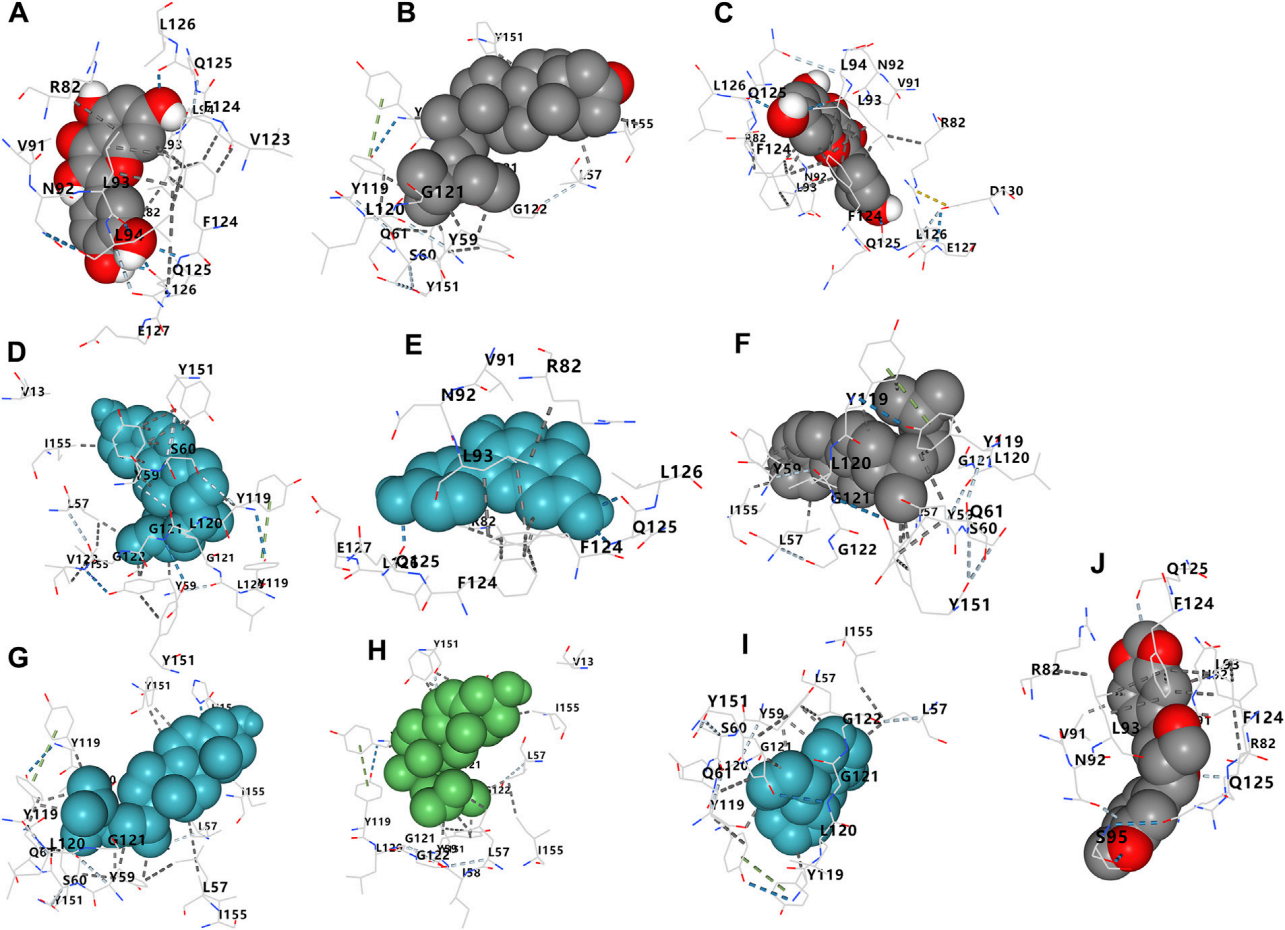
Three-dimensional map of the binding of compounds. **(A)** Quercetin, **(B)** NSC63551, **(C)** isorhamnetin, **(D)** beta-sitosterol, **(E)** kaempferol, **(F)** and CLR, **(G)** campest-5-en-3beta-ol, **(H)** Isofucosterol, **(I)** matrine, **(J)** sesamin with TNF-α.

A total of 10 compounds were found to bind to TNF-α to varying degrees (Table 3). Vina scores indicate binding capacity, with lower scores indicating a stronger and more stable interaction between the compound and acceptor. Sesamin had the highest Vina scores with TNF-α, with the strongest and most consistent affinity for TNF-α among the 10 compounds evaluated. The 3D map of TNF-α and the molecular binding of the compounds to TNF-α are shown in Figures 8, 9, respectively.

**TABLE 3:** Molecular docking parameters and results of the binding of *Semen cuscutae* (SC) active ingredients to TNF-α.

Molecule ID	Molecule name	Vina score	Cavity size	Center^a^			Size^b^		
				x	y	z	x	y	z
MOL001558	Sesamin	−9.2	1,253	−5	82	28	23	23	23
MOL000184	NSC63551	−9.1	233	−12	69	18	24	24	24
MOL000354	Isorhamnetin	−8	1,253	−5	82	28	22	22	22
MOL000358	Beta-sitosterol	−8.6	233	−12	69	18	25	25	25
MOL000422	Kaempferol	−7.8	1,253	−5	82	28	21	21	21
MOL005043	Campest-5-en-3beta-ol	−8.8	233	−12	69	18	25	25	25
MOL005440	Isofucosterol	−9.1	233	−12	69	18	25	25	25
MOL005944	Matrine	−8.1	1,253	−5	82	28	19	19	19
MOL006649	Sophranol								
MOL000953	CLR	−7.9	233	−12	69	18	25	25	25
MOL000098	Quercetin	−8.4	1,253	−5	82	28	21	21	21

### 3.6 Sesamin affects human trophoblast proliferation and apoptosis

**FIGURE 10:**
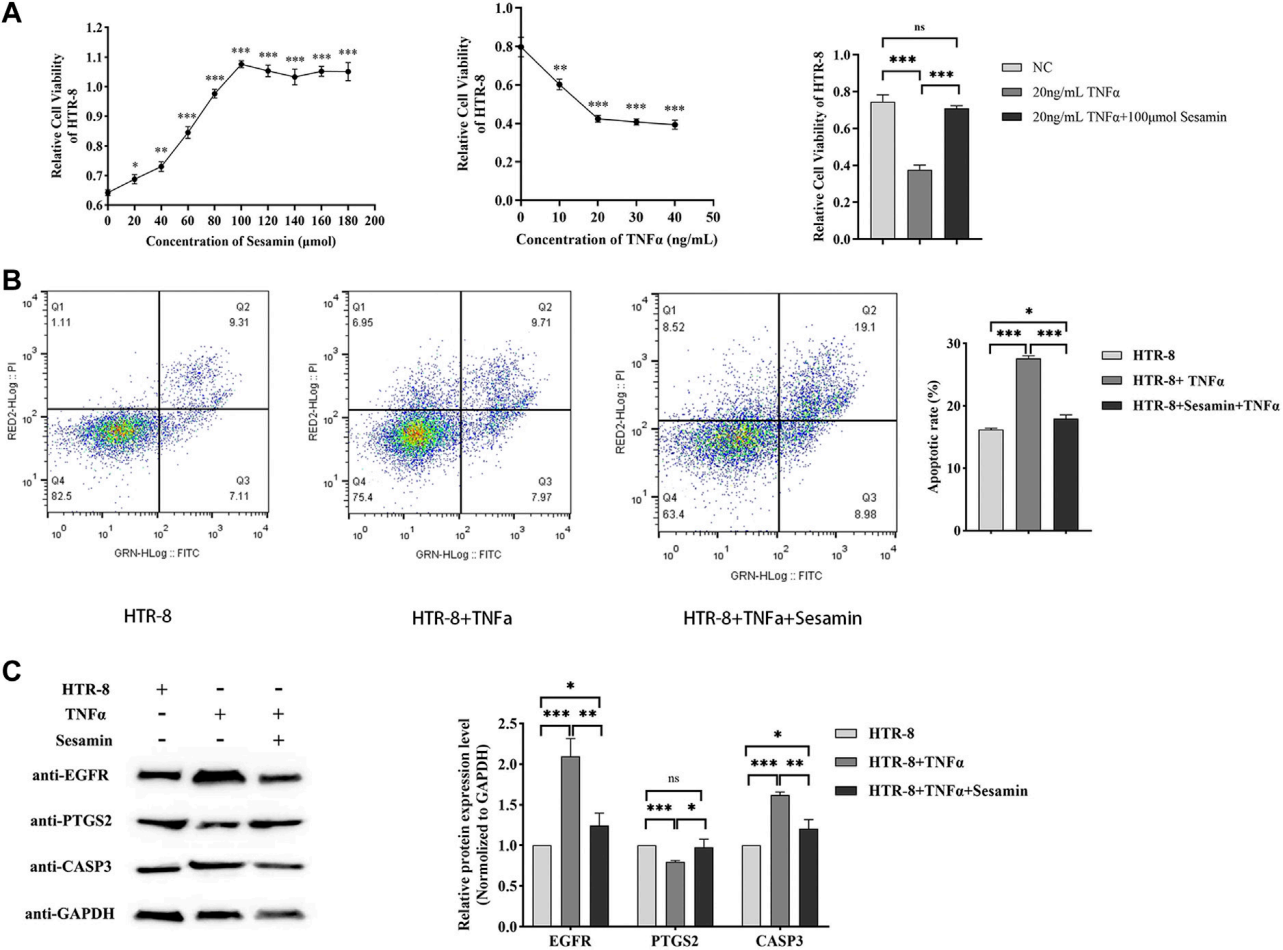
Sesamin can promote proliferation and inhibit apoptosis in human trophoblasts by downregulating EGFR and CASP3 expression and upregulating PTGS2 expression **(A)**. CCK-8 assay was used to detect cell proliferation **(B)**. Flow cytometry was performed to assess the cell apoptotic rate **(C)**. Western blot analysis was used to detect EGFR, CASP3, and PTGS2 protein levels at 24 h after treating the cells with sesamin and TNF-α. GAPDH was used as an internal control. All data are presented as mean ± SDs. *p < 0.05; **p < 0.005; and ***p < 0.001.

To investigate the effects of sesamin and TNF-α on the proliferation of HTR-8/SVneo cells, CCK-8 and cell apoptosis assays were performed by treating the cells with sesamin and TNF-α. Additionally, hub gene (EGFR, CASP3, and PTGS2) expression was analysed using Western blot analysis. The results demonstrated that different concentrations of sesamin can enhance cellular activity, among which 100 µmol of sesamin has the best effect. TNF-α can reduce cellular activity; the treatment of HTR-8 cells with 20 ng/mL of TNF-α for 24 h significantly reduced cell activity (Figure 10A). Apoptosis was significantly promoted by 20 mg TNF-α for 24 h. Sesamin reversed TNF-α-induced apoptosis (Figure 10B). The treatment of cells with 20 ng/mL of TNF-α upregulated EGFR and CASP3 expression and downregulated PTGS2 expression, whereas concurrent treatment with 100 µmol of sesquiterpenes decreased the expression levels of EGFR and CASP3 and increased the expression level of PTGS2 (Figure 10C).

## 4 Discussion

RSA affects 1%–2% of couples and causes great psychological and emotional trauma to women, as well as financial burden. The incidence of RSA is difficult to estimate due to different definitions. The known causes of RSA are complex and numerous, which include women’s age; genetic, anatomical, and chromosomal abnormalities; endocrinology; infections; placental abnormalities; smoking and alcohol consumption; and psychological factors (La et al., 2021). *Cuscuta* in the form of herbal mixtures has been known as a cure to many diseases, such as oligospermia (Wang et al., 2022), osteoporosis (Huiwen et al., 2021), premature ovarian failure (Jinying et al., 2020), sleep induction (Forouzanfar et al., 2020), habitual abortion (Wang et al., 2021b), and neurasthenia (Lu, 2016).

In this study, 10 active ingredients (including flavonoids, glycosides, alkaloids, and steroids) from SC play an important role in the treatment of RSA. Hyperoside is a flavonoid, which belongs to polyphenolic compounds. Some studies have reported that hyperoside has a protective effect on pregnancy loss in rats by increasing autophagy and inhibiting inflammation. This effect may be related to the inhibition of mTOR/S6K (Wei et al., 2020). Aberrant dendritic cell (DC) activities and differentiation have been identified to be associated with RSA. It has been reported that baicalin attenuates embryonic resorption in RSA mice by inhibiting STAT5-ID2 expression and reversing the expression of traditional DCs to plasmacytoid DCs and functional molecules (Lai et al., 2022). Increasing evidence shows that T-helper cell imbalance is associated with RSA. One study suggested that CD4 T-helper cells and circulating/uterine natural killer (NK) cell populations are associated with RM and implantation failure (Bansal et al., 2011). Th1/Th2 imbalance can lead to recurrent miscarriage (Saito et al., 2010), as confirmed by Niu Y, and matrine can regulate the balance through NF-κB signalling (Niu et al., 2017). Some studies have also confirmed that matrine can regulate Th1/Th2 balance through upregulated IFN-γ and downregulated TGF-β expression (Zhang et al., 2020). Overall, these compounds are related to multiple proteins and signalling pathways, which are of significant potential research value in RSA and deserve further investigation.

In addition, as shown in Figure 2, the results of the PPI indicated that 128 target proteins are not independent of each other but are connected and interact with each other. TNF, IL-6, TP53, IL1B, EGFR, JUN, CASP3, ESR1, PPARG, and PTGS2 were the top 10 hub genes. As shown in Figure 3, many compounds may be involved in RSA by regulating multiple target genes, such as TGFβ1, PTGS2, KCNH2, and OPRM1. These genes are differentially expressed, as verified externally by the GEO database. As shown in Figure 4, only the expression of EGFR (*p* < 0.001) and PTGS2 (*p* < 0.05) was statistically differentiated in GSE165004, while in GSE43256, there were different expression levels among TNF, PTGS2, and CASP3.

Enrichment analysis of GO and KEGG showed that 57 biological functions and 82 signalling pathways were involved in the development of RSA, which may be the mechanism of treatment of recurrent abortion by SC. The FOxO signalling pathway regulates a variety of physiological events, such as cell cycle control, apoptosis, resistance to oxidative stress, and glucose metabolism. There are no reports on the relationship between the FOxO pathway and RSA. The FOxO signalling pathway may have some relationship with RSA in the above aspects, but further studies are needed.

The TNF signalling pathway is a classic signalling pathway involved in the onset and development of many diseases, expression of inhibiting TNF-α, and the symptoms of anti-TNF-α antibody therapy that alleviate inflammatory diseases (Kang et al., 2021). The levels of a variety of cytokines are altered in RSA patients, such as TNF-α and the interleukin family, which may be involved in the occurrence of RSA (Zhang et al., 2016). Studies have confirmed that IL-4 and IL-10 promote embryonic development and placentation, whereas IFN-c and TNF-α inhibit trophoblast growth and differentiation and have the opposite effect (Daher et al., 2003). Prostaglandin–endoperoxide synthase (PTGS), also known as COX2, whose expression is induced by TNF-α, is responsible for prostanoid biosynthesis involved in inflammation and mitogenesis. Namita Singh showed that TNF-α induced COX2 expression in endometrial curettage tissue, leading to spontaneous abortion in *Chlamydia trachomatis*-infected pregnant women (Singh et al., 2020). However, animal experiments showed that the serum and embryo tissue COX2 expression level of mice in the recurrent abortion group was significantly lower than that in the control group (Hua et al., 2013), thus revealing contradictory results between the two. The results of our experiments showed that PTGS2 expression was decreased after TNF-α treatment of cells, which was consistent with the results of former animal experiments. CASP3 is involved in the occurrence of miscarriage, and a study has shown that plastics and nanoplastics can activate Bcl-2/cleaved-caspase-2/cleaved-caspase-3, leading to excessive apoptosis in human trophoblast cells and mouse placental tissues, further inducing miscarriage (Wan et al., 2024).

The active components of SC can regulate sex hormone abnormalities and reduce oxidative stress, ER stress, and cell apoptosis by lowering the levels of TNF-α and IL-6 (Karna et al., 2019). At present, anti-TNF-α drugs, such as etanercept (ETA), infliximab (IFX), adalimumab (ADA), golimumab, and certolizumab, are widely used in the clinical treatment of inflammatory diseases, but these drugs are associated with adverse pregnancy outcomes. For example, IUGR, SA, and preterm birth are the most common adverse pregnancy outcomes associated with anti-TNF-α drugs (Mahadevan et al., 2005; Clowse et al., 2018; Chambers et al., 2019). In addition, severe infections in pregnant women, gestational diabetes mellitus, stillbirth, premature rupture of membranes, and hypertension during pregnancy have also been reported (Clowse et al., 2018; Clowse et al., 2018; Clowse et al., 2015; Marchioni and Lichtenstein, 2013). There is currently a lack of expert consensus and guidelines on the use of these drugs during pregnancy.

In our work, sesamin with good OB and DL properties was selected from SC. Moreover, it had the strongest binding ability to the TNF-α molecule. Sesamin originates from the western regions of ancient China and belongs to the linseed seed family (Kamal-Eldin et al., 2011). Sesamin can significantly reverse intestinal ischaemia-reperfusion-increased IL-6, TNF-α, and IL-1β levels and exerts anti-inflammatory and antioxidant effects through the Nrf2/HO-1/NQO1 signalling pathway (Wang et al., 2021c). *In vitro* model studies suggest that sesamin has a protective effect on cartilage destruction induced by the combination of tumour necrosis factor-α and ondomasin-M (Khansai et al., 2016). These results suggest that sesamin may inhibit TNF-α and may be a promising alternative treatment for patients with RSA. Our experiments confirmed that sesamin promotes human trophoblast proliferation and inhibits apoptosis, which may be achieved by the downregulation of EGFR and CASP3 and upregulation of PTGS2. However, specific research mechanisms need to be explored further experimentally.

Taking these findings into account provides a theoretical basis for further development of natural TNF inhibitors (TNFis) and the development of new anti-inflammatory immune drugs using SC as a base substance. However, this paper has some limitations: 1) the absorption of compounds in humans is not limited to OB; 2) interactions between active ingredients are not taken into account; and 3) the above mechanism needs further experimental verification.

## Data Availability

The raw data supporting the conclusions of this article will be made available by the authors, without undue reservation.
